# Coexistence Demonstration and Wavelength Dependency Analysis of S-Band CV-QKD Signal with Fully Loaded C+L-Band DWDM Signals

**DOI:** 10.3390/e27010045

**Published:** 2025-01-08

**Authors:** Tetsuo Kawakami, Hiroki Kawahara, Toshihiko Okamura, Wakako Maeda

**Affiliations:** Advanced Network Research Laboratories, NEC Corporation, Kawasaki 211-8666, Kanagawa, Japan; kawahara-hiroki@nec.com (H.K.); t_okamura@nec.com (T.O.); wakako.maeda@nec.com (W.M.)

**Keywords:** quantum cryptography, quantum key distribution, continuous-variable QKD, wavelength division multiplexing, spontaneous Raman scattering light

## Abstract

We demonstrated the coexistence of an S-band CV-QKD signal with fully loaded C+L-band classical signals for the first time. The secret key rate of the S-band QKD system was 986 kbps with the C+L-band WDM signals transmitted through a 20 km G.654.E fiber link. We also revealed that the S-band CV-QKD performance limiting factor under the C+L-band WDM condition is the spontaneous Raman scattering light similar to the C-band CV-QKD performance limiting factor, confirming the validity of estimating the wavelength dependency of the secret key rate under the WDM condition from the fiber loss and the spontaneous Raman scattering light power. These results show that the CV-QKD performance under the C+L band WDM conditions becomes comparable to that under the C-band WDM conditions by wavelength design in the S-band.

## 1. Introduction

Quantum key distribution (QKD) in combination with one-time pad encryption provides unconditional secure data transmission [1]. QKD is classified into Discrete Variable (DV) and Continuous Variable (CV) QKD. While DV-QKD requires expensive and specialized photon detectors, CV-QKD can detect a quantum signal using a standard coherent receiver. Coherent communication technologies have high maturity and commonly available optical devices, which leads to cost-effective QKD devices. CV-QKD is more robust against stray light noise than DV-QKD, making it possible to coexist with quantum and classical signals through wavelength division multiplexing (WDM). Unlike DV-QKD, CV-QKD does not require a dedicated fiber [2]. In order to develop widespread QKD networks by remarkably reducing the costs of both QKD devices and optical fibers for the QKD path, it is highly desirable to integrate a CV-QKD system into an already deployed optical network.

Recently, much research has demonstrated the WDM transmission of the QKD signal with THz-order classical signals [3,4,5,6,7,8,9]. Because current WDM transmission systems are trying to use more wide-range bands to keep up with the increasing traffic demands, the previous QKD coexistence demonstrations with single-band WDM signals are not sufficient to emulate realistic WDM conditions. In our recent work [10], we demonstrated the coexistence of a C-band CV-QKD signal with multi-band (C+L-band) classical signals, revealed that the Spontaneous Raman Scattering (SpRS) light induced by the WDM signals significantly degraded the C-band CV-QKD performance. This result suggests that allocating QKD wavelength out of the C+L-band will improve the CV-QKD performance by reducing the SpRS light power. Therefore, it is necessary to establish the QKD wavelength design method under the multi-band WDM condition by investigating the wavelength dependency of the CV-QKD performance, including the out-band of the WDM signals. Because previous research [9] showed that the CV-QKD performance of an S-band is better than that of an L-band with C-band DWDM signals, it is desirable to clarify the wavelength dependency of the S-band QKD under the C+L-band WDM condition in detail to expand the CV-QKD application area and realize widespread QKD networks.

In this paper, we demonstrated the coexistence of an S-band (1480 nm) CV-QKD signal with fully loaded C+L-band (1529~1609 nm) classical signals for the first time. The total WDM bandwidth is 9.30-THz (124 × 75-GHz channels), and the total WDM power is +19 dBm, simulating the realistic condition of already deployed WDM systems. The Secret Key Rate (SKR) of the S-band QKD system was 986 kbps with the C+L-band WDM signals over a 20 km G.654.E fiber link. We also revealed that the S-band CV-QKD performance limiting factor under the WDM condition is the SpRS light similar to the C-band CV-QKD performance limiting factor, confirming the validity of estimating the wavelength dependency of the S-band SKR from the fiber loss and the SpRS light power.

## 2. Experimental Setup

**Figure 1:**
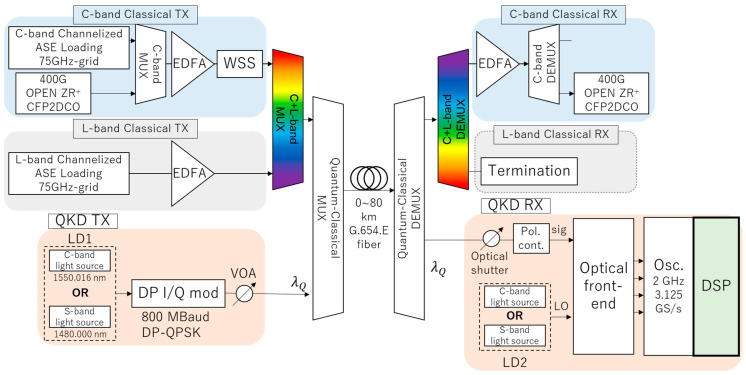
Experimental setup.

The experimental setup is shown in Figure 1. The QKD TX has two coherent light sources as LD1. The transmitter chooses one of the two light sources: a C-band light source tuned to 1550.016 nm or an S-band light source tuned to 1480.000 nm. The CV-QKD modulation is implemented using a Dual Polarization (DP)-I/Q-modulator, in which $f_{p}$ = 800 MBaud Quadrature Phase-Shift Keying (QPSK) signals drive each polarization. The first (X-pol) and the second polarization (Y-pol) are used for the quantum and a reference signal, respectively. We use the polarization multiplexed QPSK reference signal for clock timing and phase recovery of the quantum signal. We set the power of the reference signal to +30 dB higher than that of the quantum signal. To avoid polarization crosstalk from the reference signal to the quantum signal, we set the time difference 12fp = 625 ps between the quantum and reference signals.

The QKD RX has two coherent light sources as LD2 for the local oscillator (LO) and chooses the same wavelength light source as the QKD TX. The linewidth of the two LO light sources is 10 kHz. The optical front-end consists of a DP-90-degree hybrid and four balanced receivers with a bandwidth of 1.6 GHz. The transmitted signals are digitized using a real-time oscilloscope. Four received signals are processed using a Digital Signal Processing (DSP) algorithm to demodulate the quantum signal. We measured the noise clearance between shot noise and electronic noise as $v_{el}$; the $v_{el}$ of the C-band (S-band) QKD RX was 0.160 (0.097). We also measured the quantum efficiency of the QKD RX as $\eta_{2}$; the $\eta_{2}$ of the C-band (S-band) QKD RX was 0.35 (0.29).

**Figure 2:**
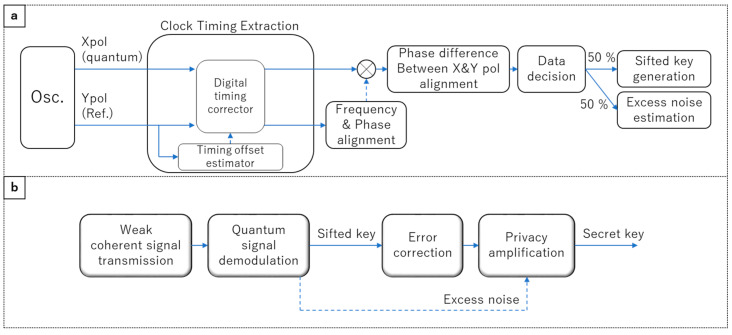
(**a**) Flow of the digital signal processing for the quantum signal demodulation. (**b**) Flow of the secret key distillation. The solid and dashed arrows represent the mainstream and substream of DSP, respectively.

We carried out quantum signal demodulation via offline DSP, as shown in Figure 2a. This includes clock timing extraction, alignment of the frequency and the phase difference between LD1 and LD2, and alignment of the phase difference between the quantum and reference signals. The clock timing extraction is achieved using the feedforward-based method [11]. We estimate the timing offset of the quantum signal from the reference signal and control a digital timing corrector to adjust the clock timing. The optical shutter at the input of the QKD RX is used for shot noise calibration. When the shutter is open, the detected digital signals are demodulated by the DSP, and the variance of the quantum signal is obtained. When the shutter is closed, the detected digital signals pass through the DSP, and the variance of the shot noise is obtained. During one shutter cycle, we can measure one excess noise by comparing the variance of the shot noise and that of the quantum signal. We set the variance calculation block size to 10^4^ bit. The sifted key generation efficiency $\eta_{e}$ is 0.25 because we use half of the measurement time to demodulate the quantum signal and the other half to calibrate the shot noise, and we use 50% of the demodulated quantum signal data for sifted key generation and the other 50% for excess noise estimation. The QKD TX and RX share the secret keys after applying error correction and privacy amplification to the sifted keys, as in Figure 2b. Note that excess noise critically affects the CV-QKD performance because the parameter is used to estimate the amount of information leaking.

**Figure 3:**
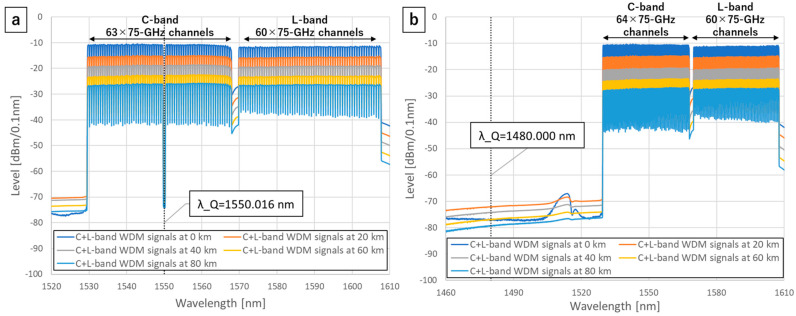
(**a**) C+L-band WDM signals with the C-band Quantum–Classical MUX filter. (**b**) C+L-band WDM signals with the S-band Quantum–Classical MUX filter.

We co-propagate a C-band or S-band CV-QKD signal with C-band 63λ or 64λ and L-band 60λ 75-GHz WDM classical signals over 0, 20, 40, 60, and 80 km G.654.E fiber links. The spectrums of the WDM classical signals with the Quantum–Classical multiplexing (MUX) filters are in Figure 3. The classical signals are generated by using channelized Amplified Spontaneous Emission (ASE) loadings. The classical signals are amplified by an Erbium-Doped Fiber Amplifier (EDFA) and then launched into a transmission line. Although the power of the WDM classical signals should be small to minimize the effect of the QKD performance, the post-FEC BER of the classical signals must be zero for the coexistence of quantum and classical signals. For measuring the BER of the classical signals, we multiplexed a C-band CFP2-DCO transceiver signal with the WDM signals and set each C-band/L-band with a launch power of +16 dBm and a total launch power of +19 dBm (−1.9 dBm/ch). We set the transceiver’s wavelength to 1532.779 or 1564.572 nm, which is the 5λ inside the top or the bottom of the C-band. We confirmed that the post-FEC BERs are zero over 0~80 km, which means there is no degradation of the quality of classical signals due to WDM. We use an add/drop filter for MUX and demultiplexing (DEMUX) the quantum and the classical WDM signals. The C-band Quantum-Classical MUX filter exhibits a band-stop profile with a 3 dB bandwidth of 87 GHz and an isolation of 25 dB within the quantum signal band, sufficiently suppressing QKD in-band ASE lights. Similarly, the C-band Quantum–Classical DEMUX filter has a band-pass profile with the same bandwidth and an isolation of 40 dB, effectively attenuating out-of-band classical signals. We use S/C/L-band 3port WDM filters as the S-band Quantum–Classical MUX and DEMUX filters. The central wavelength of these S-band MUX and DEMUX filters is 1490 nm, the bandwidth is 5.4 THz (1490±20 nm), and the isolation is 30 dB.

## 3. Results

### 3.1. Analyzation of the QKD In-Band Noise Power

**Figure 4:**
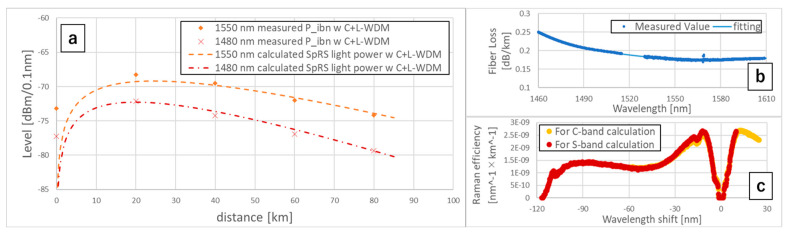
(**a**) Measured QKD in-band noise powers $P_{ibN}$ and calculated SpRS light powers with the C+L-band WDM. (**b**) Measured fiber loss. (**c**) Measured Raman efficiency of the fiber.

We measured the noise power of the $\lambda_{Q}$ as the QKD in-band noise power $P_{ibN}$. The $P_{ibN}$ of $\lambda_{Q}$ = 1550.016, 1480.000 nm under the C+L-band WDM condition at the transmission distances 0, 20, 40, 60, and 80 km were plotted in Figure 4a. We plotted the loss of the G.654.E fiber in Figure 4b. We can see that the $P_{ibN}$ of $\lambda_{Q}$ = 1480.000 nm was around 5 dB lower than the $P_{ibN}$ of $\lambda_{Q}$ = 1550.016 nm, while the fiber loss of $\lambda_{Q}$ = 1480.000 nm (0.218 dB/km) became worse than the loss of $\lambda_{Q}$ = 1550.016 nm (0.184 dB/km). We obtained the Raman efficiency of the fiber [12,13] by measuring the power of the SpRS light induced from a 1550 nm 75 GHz channelized ASE pump light, plotted in Figure 4c. The wavelength shift in Figure 4c refers to the wavelength difference from the wavelength of the pump light. From Figure 4c, we confirmed that the Raman efficiency decreases sharply below the wavelength shift of −110 nm. We calculated the SpRS light power of $\lambda_{Q}\;$= 1550.016 nm, induced by the C+L-band WDM signals by the Raman efficiency of wavelength, shift from −21~+57 nm, and the SpRS power of $\lambda_{Q}$ = 1480.000 nm by the efficiency of the wavelength shift from −120~−49 nm. The calculated SpRS light powers were plotted in Figure 4a on the dotted curves. Although the calculated SpRS light power and the measured $P_{ibN}$ differed at 0 km because of the insufficient ASE suppression of our system, they were well matched in 20~80 km, which indicates that SpRS lights dominate the QKD in-band noise of our C-band and S-band QKD systems, the impact of the four-wave mixing (FWM) is negligible over 20 km fiber links, corresponding to previous work [14].

### 3.2. Analysis of the Excess Noise at the QKD RX

**Figure 5:**
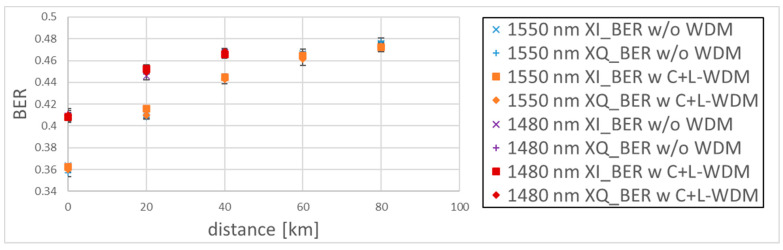
BERs of the C-band and S-band QPSK quantum signal.

We demodulated the quantum signal in the following four conditions: $\lambda_{Q}\;$= 1550.016 nm without WDM, $\lambda_{Q}$ = 1550.016 nm with the C+L-band WDM, $\lambda_{Q}$ = 1480.000 nm without WDM, and $\lambda_{Q}$ = 1480.000 nm with the C+L-band WDM. The power of the quantum signal at the QKD TX was −73.7 dBm, corresponding to the mean photon number of 0.4. We succeeded in demodulating the C-band quantum signal at the transmission distances 0, 20, 40, 60, and 80 km, but our QKD RX could not demodulate the S-band quantum signal at the transmission distance above 60 km, even without WDM, owing to the high loss of the S-band. We plotted the BERs of the C-band and S-band quantum signal in Figure 5 with the standard deviations, showing no significant difference between the BER without WDM signals and the BER with WDM signals. The BER of the S-band quantum signal was worse than that of the C-band quantum signal because of the high loss of the S-band.

**Figure 6:**
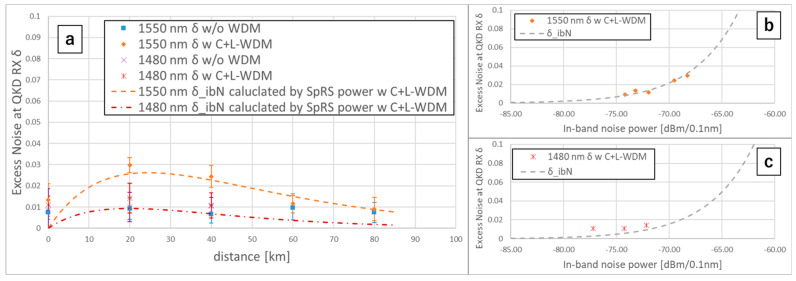
(**a**) Measured excess noises δ and theoretical excess noises $\delta_{ibN}$ calculated by the SpRS light powers. (**b**,**c**) Measured excess noises δ plotted by the QKD in-band noise powers $P_{ibN}$ with the C+L-band WDM.

We measured the δ, the excess noise at the QKD RX, by comparing the variance of the demodulated quantum signal and that of the shot noise in the above four conditions. We measured the δ five times on the variance calculation block size of $10^{4}$ bit and plotted the mean δ with the standard deviations in Figure 6a. In Figure 6b,c, under the C+L-band WDM condition, we plotted the measured δ of $\lambda_{Q}$ = 1550.016, 1480.000 nm by the $P_{ibN}$ on the dots and plotted the theoretical $\delta_{ibN}$ by the $P_{ibN}$ on the dotted curves. The $\delta_{ibN}$ is calculated only by the QKD in-band noise power [15], which is given as follows:

$$\delta_{ibN}=\eta_{2}\times \frac{P_{ibN}}{\frac{hc}{\lambda_{Q}}\times f_{p}}$$

where h is the Planck constant, and c is the velocity of light. In Figure 6b,c, the measured δ with the WDM condition generally corresponded to the theoretical $\delta_{ibN}$, suggesting that under the C+L-band WDM condition, the QKD in-band noise power dominantly affects the excess noise; other factors such as shot noises generated in C-band or L-band WDM signals [16] are negligible for the excess noise. In other words, we clarified that the S-band CV-QKD performance limiting factor under the C+L-band WDM condition is the QKD in-band noise power, dominated by SpRS lights, similar to the C-band CV-QKD performance limiting factor. From this suggestion, we can say that $\delta_{ibN}$ calculated by the SpRS light power is the best δ under the WDM condition because the excess noise of the ideal QKD TX and RX is zero except for the excess noise caused by WDM classical signals. We plotted the $\delta_{ibN}$ calculated by the SpRS light power with the C+L-band WDM (as shown by the dotted curves in Figure 6a) for estimating the wavelength dependency of the best SKR.

### 3.3. Secret Key Rate of Our CV-QKD System

We use the CV-QKD security proof against collective attacks [17]. The SKR is given as follows:

$$SKR=f_{p}\eta_{e}\left\{\beta I\left(A|B\right)-\chi \left(E|B\right)\right\}$$

where β is the reconciliation efficiency, $I\left(A|B\right)$ is the mutual information between the QKD TX and RX, and χ(E|B) is the Holevo information between Eavesdropper and the QKD RX. We calculated $I\left(A|B\right)$ and χ(E|B) by the mean photon number at the QKD TX n, the transmittance of the channel $\eta_{1}$, the quantum efficiency of the QKD RX $\eta_{2}$, the noise clearance of the QKD RX $v_{el}$, and the excess noise of the quantum channel ξ. Note that in the security proof [17], SKR is calculated not by the excess noise at the QKD RX δ but by the excess noise of the quantum channel ξ. We obtained the ξ by dividing the measured δ by the channel transmittance. The SKRs of our previous work [18] were much higher than those in this work because we used δ to calculate SKRs against a specific collective attack (e.g., an Entangling Cloner attack [2]). The above parameters in this work are as follows: β was 0.95; n was 0.4; and the $\eta_{1}$ values of 1550.016 nm (1480.000 nm) at 0, 20, 40, 60, 80 km were 1.00, 0.43, 0.18, 0.078, 0.034 (1.00, 0.37, 0.13, 0.049, 0.018), respectively. We obtained the ξ by dividing the measured δ (Figure 6a) by the channel transmittance.

**Figure 7:**
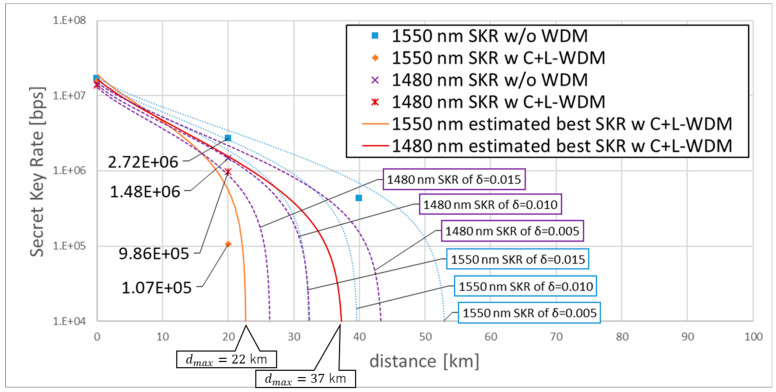
SKRs of $\lambda_{Q}=1480,\;1550$ nm without/with the C+L-band WDM.

We plotted the SKRs (as shown in Figure 7). The six dotted curves in Figure 7 are the SKRs of $\lambda_{Q}$ = 1550.016, 1480.000 nm by the three fixed δ (0.005, 0.010, 0.015), showing that excess noise critically affects the CV-QKD performance. The dots in Figure 7 are the SKRs by measured δ of Figure 6a. At the transmission distance of 20 km, the SKR of $\lambda_{Q}$ = 1550.016 nm without WDM was $2.72\times 10^{6}$ bps and degraded to $1.07\times 10^{5}$ bps by the C+L-band WDM signals, the SKR of $\lambda_{Q}$ = 1480.000 nm without WDM was $1.48\times 10^{6}$ bps and degraded to $9.86\times 10^{5}$ bps by the C+L-band WDM signals. This result showed the feasibility of our S-band CV-QKD system in distributing secret keys even under the C+L-band 9.30-THz wide bandwidth and +19 dBm high-power WDM conditions. Under the WDM condition, the S-band SKR was much higher than the C-band SKR, showing the advantage of the S-band CV-QKD under the WDM condition. The two solid curves in Figure 7 are the best SKRs estimated by the $\delta_{ibN}$, which is calculated by the SpRS light power, showing that the maximum secret key distributing distance $d_{max}$ of $\lambda_{Q}$ = 1550.016 nm under the C+L-band WDM condition was around 22 km, and the $d_{max}$ of $\lambda_{Q}$ = 1480.000 nm is around 37 km.

## 4. Discussion

**Figure 8:**
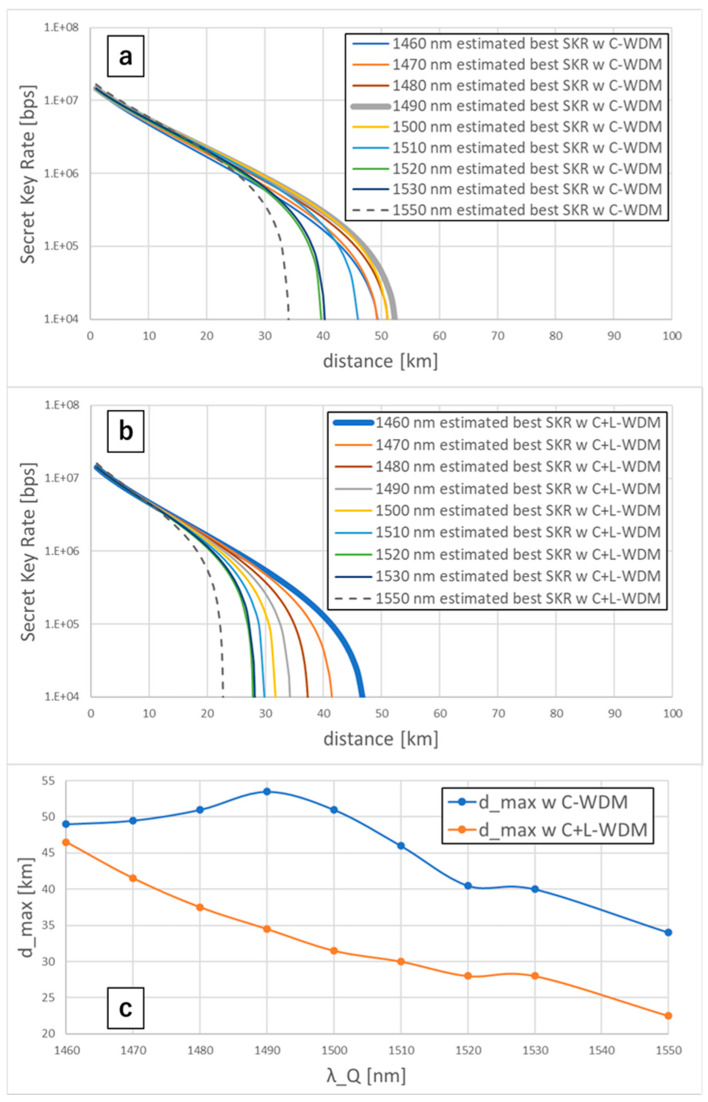
(**a**) Estimated best SKRs of $\lambda_{Q}=1460\sim 1530,\;1550$ nm with 64λ + 16 dBm C-band WDM. (**b**) Estimated best SKRs of $\lambda_{Q}=1460\sim 1530$, 1550 nm with 124λ + 19 dBm C+L-band WDM. (**c**) Maximum secret key distributing distance $d_{max}$ of $\lambda_{Q}=1460\sim 1530,\;1550$ nm with the C-band/C+L-band WDM.

To establish the QKD wavelength design method under the WDM condition, we estimated the best SKRs in the S-band ($\lambda_{Q}$ = 1460~1530 nm) from the wavelength dependency of the fiber loss and the $\delta_{ibN}$ calculated by the SpRS light power under the C-band WDM condition of the +16 dBm 64 × 75 GHz channels and the C+L-band WDM condition of the +19 dBm 124 × 75 GHz channels. We plotted the best SKRs estimated by the transmission distance (as shown in Figure 8a,b). We measured the $d_{max}$ as the distance at which the SKRs are below $1\times 10^{4}$ bps. We plotted the $d_{max}$ of $\lambda_{Q}$ = 1460~1530 nm using the $\lambda_{Q}$ (as shown in Figure 8c). In Figure 8c, the $d_{max}$ under the C-band WDM condition increased with the shorting of the $\lambda_{Q}$, reaching the maximum value at 1490 nm and then decreasing. On the other hand, the $d_{max}$ under the C+L-band WDM condition monotonically increased with the shorting of the $\lambda_{Q}$. This shows that the optimal QKD wavelength depends on classical WDM conditions. When the total WDM power is high (e.g., under C+L-band WDM conditions), the intense SpRS light induced by the WDM signals dominantly affects the SKR rather than the high fiber loss, so the best SKR can be obtained by allocating the QKD wavelength to minimize the SpRS light power. When the total WDM power is not high (e.g., under C-band WDM conditions), the best SKR can be obtained by controlling the trade-off relation between the SpRS power and fiber loss. The difference between $d_{max}$ under the C+L-band WDM condition and that of the C-band WDM condition decreased with the shorting of the $\lambda_{Q}$, and they almost corresponded at 1460 nm, suggesting that the SpRS light induced by the L-band WDM signals decreased with shorting the $\lambda_{Q}$ and became almost zero at 1460 nm. This suggestion is supported by the results illustrated in Figure 4c, which shows that the Raman efficiency of the fiber decreases sharply below the wavelength shift of −110 nm. We confirmed the advantage of the S-band QKD compared to the C-band QKD under the C+L-band WDM condition; using QKD wavelength design, we can make the CV-QKD performance under multi-band (C+L-band) WDM conditions comparable to the performance under single-band (C-band) WDM conditions.

## 5. Conclusions

We demonstrated the coexistence of the S-band CV-QKD signal with fully loaded C+L-band classical signals for the first time. The total WDM bandwidth is 9.30 THz, and the total WDM power is +19 dBm, simulating the realistic condition of already deployed WDM systems. The SKR of the C-band/S-band QKD system was 107 kbps/986 kbps with the C+L-band WDM signals over a 20 km G.654.E fiber link, showing the advantage of the S-band QKD compared to the C-band QKD under the C+L-band WDM condition. We also revealed that the S-band CV-QKD performance limiting factor under the WDM condition is the SpRS light similar to the C-band CV-QKD performance limiting factor, confirming the validity of estimating the wavelength dependency of the S-band SKR from the fiber loss and the SpRS light power. We confirm that the CV-QKD performance under the C+L band WDM conditions becomes comparable to that under the C-band WDM conditions by wavelength design in the S-band.

## Data Availability

The original contributions presented in the study are included in the article, further inquiries can be directed to the corresponding author.
